# Profiles of autophagy-related genes in esophageal adenocarcinoma

**DOI:** 10.1186/s12885-020-07416-w

**Published:** 2020-10-01

**Authors:** Lei Zhu, Lin Dong, Minghao Feng, Fugui Yang, Wenhao Jiang, Zhiyuan Huang, Fabing Liu, Lingwei Wang, Guangxue Wang, Qinchuan Li

**Affiliations:** 1 Department of Thoracic Surgery, Shanghai East Hospital, Tongji University School of Medicine, 150 Jimo Road, Shanghai, 200120 China; 2 Research Center for Translational Medicine, Shanghai East Hospital, Tongji University School of Medicine, 150 Jimo Road, Shanghai, 200120 China; 3 Department of Thoracic Surgery, Shanghai General Hospital, Shanghai Jiaotong University, School of Medicine, Shanghai, 200080 China

**Keywords:** Autophagy, Esophageal adenocarcinoma, Prognosis

## Abstract

**Background:**

Several studies have demonstrated autophagy was involved in the process of esophageal adenocarcinoma (EAC). The aim of this study was to explore autophagy-related genes (ARGs) correlated with overall survival (OS) in EAC patients.

**Methods:**

Expressions of ARGs in EAC and normal samples were downloaded from TCGA database. GO and KEGG enrichment analyses were used to investigate the ARGs bioinformatics functions. Univariate and multivariate cox regressions were performed to identify prognostic ARGs and the independent risk factors. ROC curve was established to evaluate the feasibility to predict the prognosis. Finally, the correlations between ARGs and clinical features were further explored. In addition, significantly different ARGs were verified in EAC specimens and normal esophageal mucosal tissues.

**Results:**

Thirty significantly different ARGs were selected from EAC and normal tissues. Functional enrichments showed these ARGs were mainly related apoptosis. Multivariate cox regression analyses demonstrated eight ARGs were significantly associated with OS. Among these eight genes, BECN1 (HR = 0.321, *P* = 0.046), DAPK1 (HR = 0.636, *P* = 0.025) and CAPN1 (HR = 0.395, *P* = 0.004) played protective roles in survival. Gender (HR = 0.225, *P* = 0.032), stage (HR = 5.841, *P* = 0.008) and risk score (HR = 1.131, *P* < 0.001) were independent prognostic risk factors. ROC curves showed better efficacy to predict survival using the risk score. Additionally, we found BECN1, DAPK1, VAMP7 and SIRT1 genes were correlated significantly with survival status, gender, primary tumor and tumor stage (all *P* < 0.05). The experimental results confirmed the BIRC5 was overexpressed and the ITPR1, PRKN were downregulated in the EAC tissues compared with the normal esophageal mucosal tissues (all *P* < 0.05).

**Conclusion:**

Our findings suggested that autophagy was involved in the process of EAC. Several ARGs probably could serve as diagnostic and prognostic biomarkers and may help facilitate therapeutic targets in EAC patients.

## Background

Esophageal adenocarcinoma (EAC) is a highly aggressive histologic subtype that predominates in the western countries, with a dismal 5-year survival rate of approximately 20% [1]. It is estimated the incidence of EAC was about 0.7 per 100,000 person-years and the proportions doubled from 35 to 61% over past 30 years [2, 3]. Malignancy of EAC lies in asymptomatic signs in early stage and the consequent late diagnosis. Patients with localized EAC have benefited from surgical and postoperative therapies, including neoadjuvant chemotherapy or chemoradiotherapy [4, 5]. However, managements of advanced stage or metastatic EAC only include palliative treatments and supportive care [6, 7]. In addition, the high rate of resistance to chemotherapy or radio chemotherapy also contributed to the poor prognosis [2]. Therefore, it’s imperative to identify innovative methods and biomarkers for early stage detection along with new treatment strategies. Cumulative evidences showed that autophagy was involved in the pathogenesis of EAC and may provide benefits for patients [8–10].

Autophagy is involved in the catabolic recycling for hydrolytic degradation in lysosomes to maintain hemostasis and occurs at a basal level in EAC. Autophagy serves as complex roles in EAC and correlates with Barrett’s esophagus (BE) and gastroesophageal reflux disease (GERD) [11, 12]. When exposed to chronic bile and acid reflux, autophagy plays a tumor-promoting role. In acute bile exposure and early cancer stage, it acts as a tumor-suppressor factor in EAC. Although the autophagy in the EAC has not been studied thoroughly, alterations in autophagy have been identified in several studies [11–13]. In addition, the autophagy-related genes (ARGs) and proteins including Beclin-1 and p62 have been already studied in EAC [8, 10, 14]. These studies demonstrated the critical roles of autophagy in the development of EAC and responses to therapy. However, their researches may be more reasonable if they had studied the relations between the ARGs and survival systematically. Furthermore, there also exists some contrary reports about autophagy in esophageal cancer [15, 16]. Thus, exploring the relationship between EAC and autophagy will further elucidate the mechanisms of pathogenesis and may improve the prognosis and treatment options.

In this study, we will provide a preliminary overview of the ARGs profiles between EAC and normal samples using the TCGA database, aiming at combining these genes with clinical data to provide a perspective on the future diagnostic and therapeutic options.

## Methods

### Patients samples and gene extraction

Samples of EAC and normal controls were downloaded from The Cancer Genome Atlas (TCGA) database (https://portal.gdc.cancer.gov), including gene expression profiles and clinical data. Human Autophagy Database (HADb) was used to identify 232 autophagy-associated gene at http://www.autophagy.lu, which provided the comprehensive and abundant information about autophagy from PubMed and biological public databases [17].

### Identification of DEARGs

Differentially expressed genes (ARGs) were identified by using the Wilcoxon signed-rank test. The cut-off values were determined according to the parameters, false discovery rate (FDR) < 0.05 and |log2 fold change (FC)| > 1.

### Univariate cox and multivariate proportional hazard regression

Univariate cox proportional hazard regression analysis was used to evaluate the correlations between the OS and ARGs. Then, significant prognostic factors (*P* < 0.05) were entered into multivariate cox regression to identify the independent prognostic risk factors.

### Pathway analysis and ROC analysis

The functional analyses of Gene Ontology (GO) and KEGG pathway were conducted by using R software (version 3.6.1). Top results with the false discovery rate (FDR) < 0. 05 were considered significance.

The Receiver Operating Characteristic (ROC) analysis was used to examine the sensitivity and specificity of survival prediction using the independent risk factors. The area under the ROC curve (AUC) ranges from 0.5 to 1, with near 1 indicating perfect predictive ability and 0.5 indicating without predictive ability.

### Experimental validation

To verify ARGs expression levels in EAC and normal tissues, we conducted the experimental validation in 15 EAC patients’ specimens who received esophagectomy from 2019 January to 2020 January in Shanghai East Hospital, Tongji University School of Medicine. Ten normal esophageal mucosal tissues were used as control. This study was approved by the Internal Review Board of Shanghai East Hospital, Tongji University School of Medicine.

Total RNA from EAC specimens and normal tissues was purified using RNAiso plus (Takara, Dalian, China). Complementary DNA (cDNA) was synthesized from 1 μg of total RNA using a PrimeScript® RT reagent Kit with gDNA (genomic DNA) Eraser (Takara). TB Green® Premix Ex Taq® II kit (Takara) was used to detect the indicated RNA levels on the QuantStudio Real-Time polymerase chain reaction (PCR) System (Applied Biosystems, USA) or the CFX96 Real-Time System (Bio-Rad, USA). The relative expression levels of the candidate ARGs were normalized to endogenous GAPDH (glyceraldehyde-3-phosphate dehydrogenase). The primers synthesized and by GENEWIZ company, Suzhou, China. The primers are listed in Supplementary Table S1.

## Results

### Distinct autophagy-associated genes in EAC and normal tissues

**Fig. 1:**
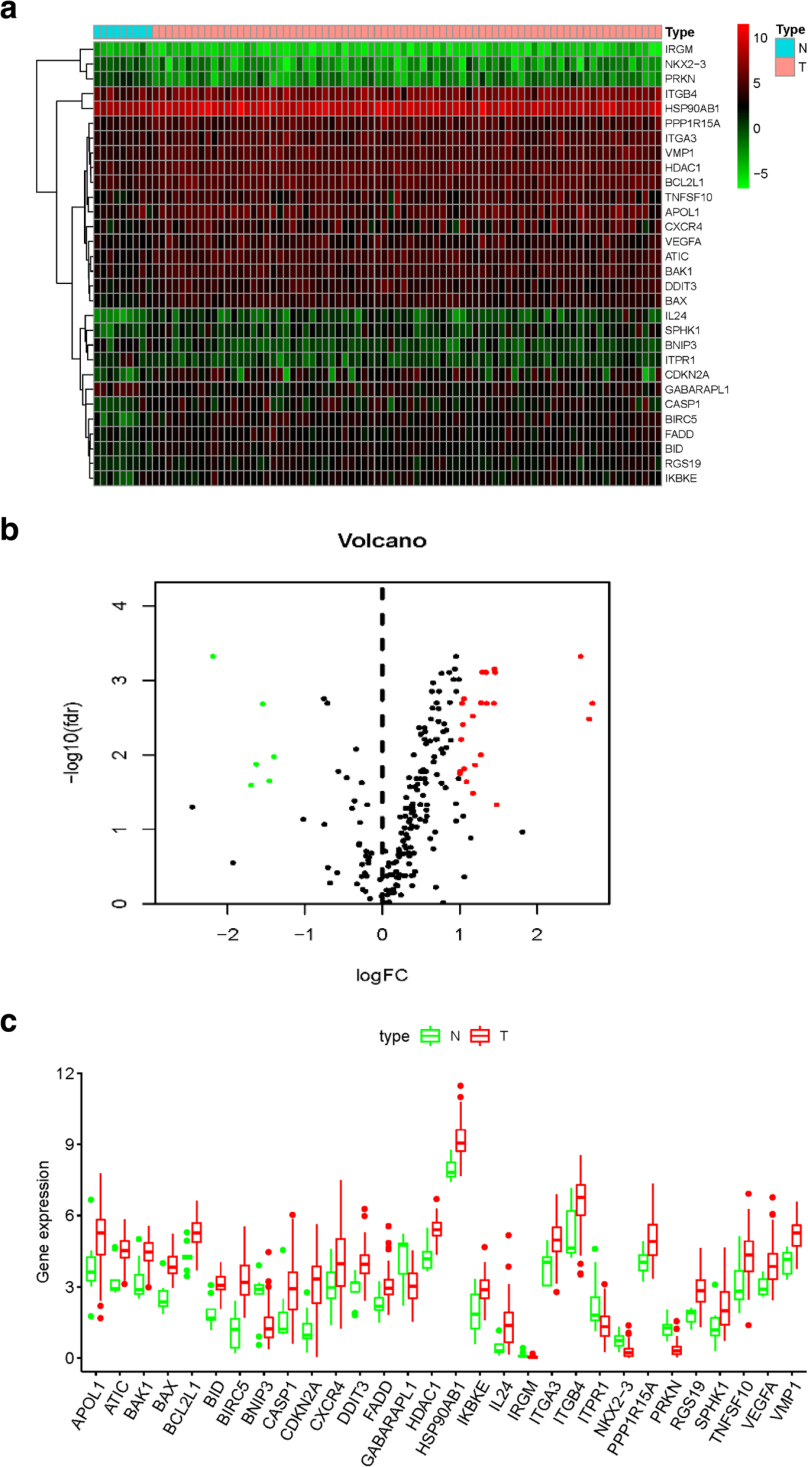
Distributions of SD-ARGs. **a** Heatmap of SD-ARGs. Green represented down-regulated genes and red represented up-regulated genes. **b** Volcano plot of SD-ARGs. Green dots represented 6 down-regulated genes; red dots represented 24 up-regulated genes. **c** The bar plot of genes in normal and tumor tissues

A total of 9 normal and 78 EAC tissues with gene expression profiles and clinical data were obtained from TCGA. There were 30 significantly different autophagy-related genes (SD-ARGs) between the normal and tumor groups. Among these genes, 6 genes were down-regulated and 24 were up-regulated in the tumor group compared with normal group (Table 1). The heatmap, volcano plot and bar plot were shown in Fig. 1a-c.

**Table 1:** SD-ARGs expression levels in EAC and normal tissue

gene	normal	EAC	logFC	*P*-value	FDR
HDAC1	21.783	44.624	1.035	0.000	0.002
BCL2L1	20.050	41.717	1.057	0.000	0.002
CASP1	4.147	10.044	1.276	0.003	0.010
GABARAPL1	21.688	8.234	−1.397	0.003	0.011
ITGB4	54.149	115.313	1.091	0.009	0.023
NKX2–3	0.702	0.241	−1.543	0.000	0.002
TNFSF10	10.653	24.494	1.201	0.004	0.014
APOL1	21.168	42.513	1.006	0.006	0.018
CXCR4	8.710	24.320	1.481	0.022	0.047
BNIP3	6.195	2.255	−1.458	0.008	0.022
VMP1	15.927	39.030	1.293	0.000	0.001
HSP90AB1	263.026	666.701	1.342	0.000	0.001
RGS19	2.514	6.916	1.460	0.000	0.001
SPHK1	1.984	4.477	1.174	0.014	0.033
FADD	4.183	8.494	1.022	0.001	0.006
PPP1R15A	17.157	35.799	1.061	0.005	0.015
BIRC5	1.741	10.314	2.566	0.000	0.000
IL24	0.379	2.488	2.715	0.000	0.002
ATIC	10.788	24.286	1.171	0.000	0.003
IRGM	0.092	0.030	−1.626	0.004	0.013
VEGFA	7.275	19.819	1.446	0.000	0.002
PRKN	1.540	0.339	−2.185	0.000	0.000
ITPR1	6.263	1.937	−1.693	0.010	0.025
DDIT3	7.140	17.326	1.279	0.000	0.002
BAX	5.593	15.299	1.452	0.000	0.001
BAK1	10.677	22.012	1.044	0.001	0.004
IKBKE	3.508	7.066	1.010	0.005	0.017
CDKN2A	1.718	10.973	2.675	0.001	0.003
BID	3.102	7.882	1.345	0.000	0.001
ITGA3	14.242	36.355	1.352	0.000	0.002

*LogFC* log fold change, *FDR* false discovery rate

### Enrichment analysis

**Fig. 2:**
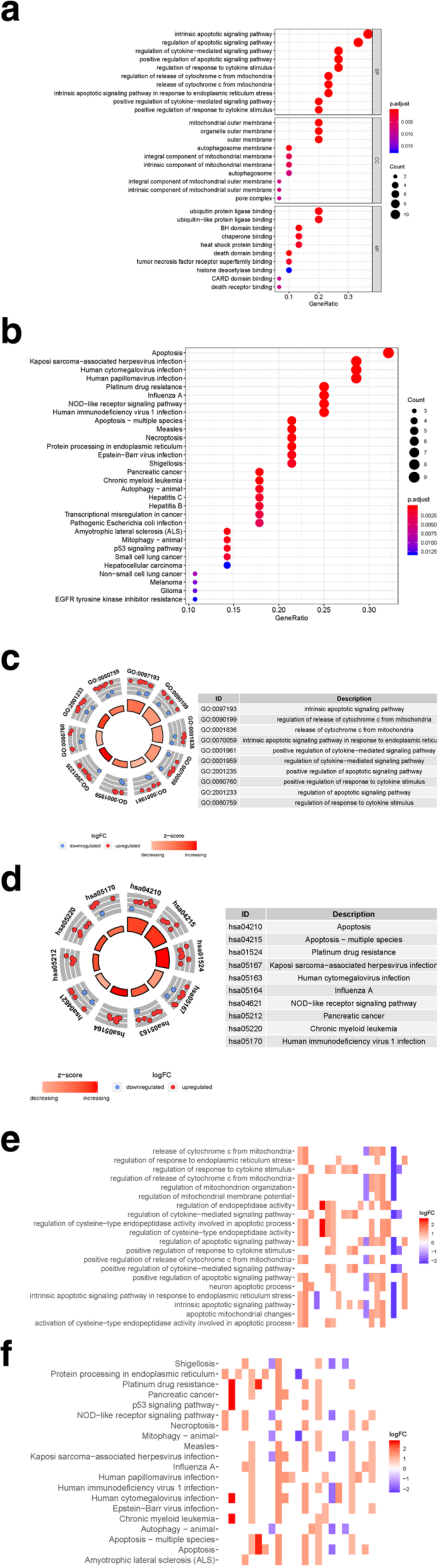
GO and KEGG enrichments of SD-ARGs **a**-**b** Showed the GO and KEGG enrichment analysis respectively. The larger bubble and darker color indicated the more significant enrichment process. **c**-**d** Enrichment pathways in the GO and KEGG circle plots respectively. The inner circle indicated Z-score. The red color represented the significant enrichment. The outer circle indicated the various pathways, in which the blue dots indicated down-regulated genes and the red was up-regulated genes. **e**-**f** The heatmaps of GO and KEGG enrichment respectively. The red color indicated the up-regulated genes and green represented down-regulated genes

GO (Gene Ontology) analysis included the biological process (BP), cellular component (CC) and molecular function (MF) categories. In the BP category, the SD-ARGs were obviously enriched in the intrinsic apoptotic signaling pathway, as well as the regulation of apoptotic signaling pathway. In the CC, the SD-ARGs were mainly enriched in the mitochondrial outer membrane process. In addition, the CC analysis also showed the SD-ARGs are involved in the autophagosome and autophagosome membrane. In the MF, the SD-ARGs were obviously enriched in ubiquitin protein ligase binding process (Fig. 2a). In the KEGG (Kyoto Encyclopedia of Genes and Genomes) analysis, the SD-ARGs were mainly enriched in the apoptosis, which was similar in the GO analysis (Fig. 2b).

In the GO circle plot, the SD-ARGs were mainly enriched in the intrinsic apoptotic signaling pathway and the regulation of cytochrome c from mitochondrion. And in the KEGG circle plot, it was also clearly showed these genes were mainly involved in the apoptotic process. Many SD-ARGs have other pathways related to the autophagy, such as the apoptosis-multiple species shown in the KEGG circle plot (Fig. 2c-d).

The GO and KEGG heatmaps revealed the SD-ARGs were enriched in the apoptotic process (Fig. 2e-f).

### Prognosis-related ARGs

**Fig. 3:**
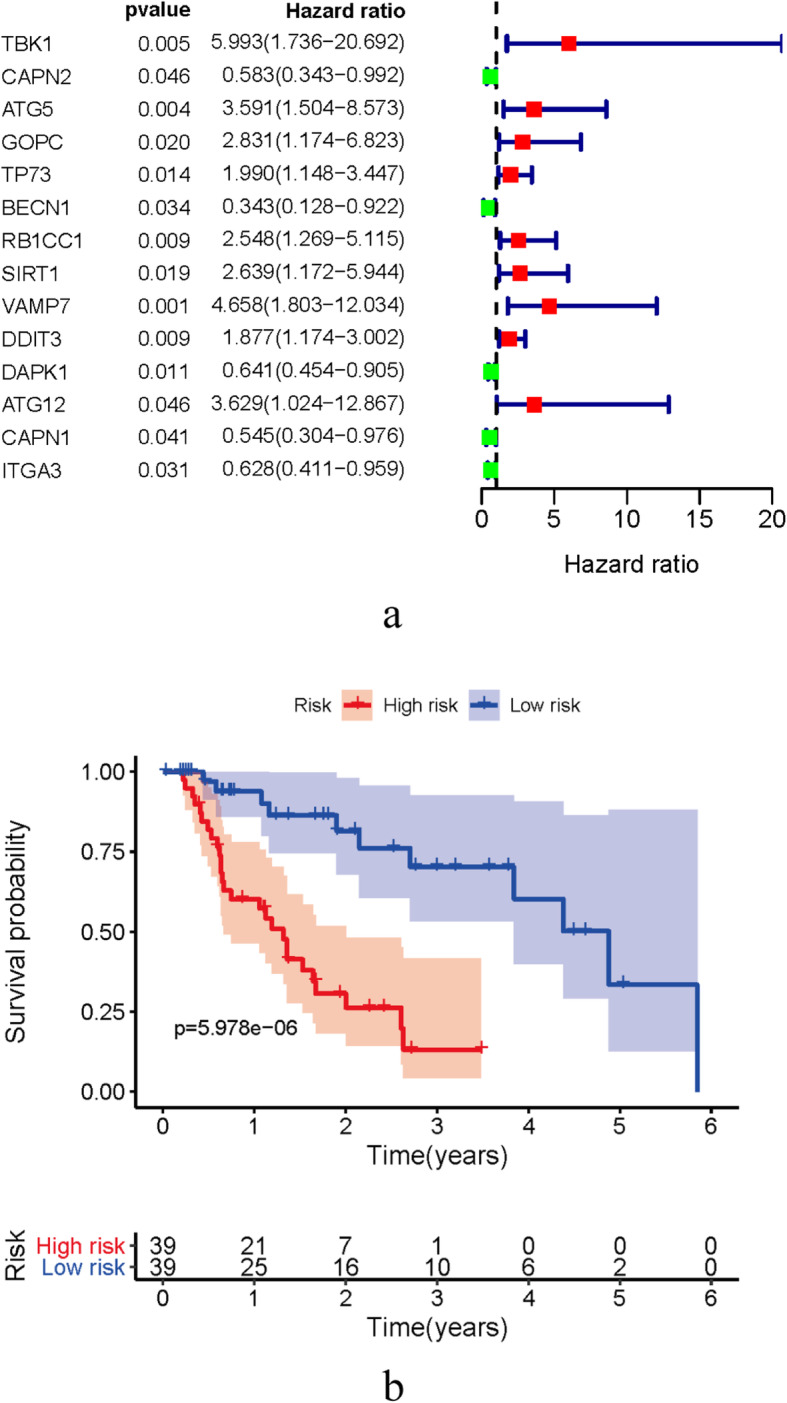
Forest plot and Kaplan-Meier curve **a** forest plot of 14 prognosis-related genes **b** Kaplan-Meier curve for OS in the high-risk and low-risk groups when stratified by the autophagy-related risk score

Univariate cox regression was used to investigate ARGs related with prognosis, 14 ARGs (TBK1, CAPN2, ATG5, GOPC, TP73, BECN1, RB1CC1, SIRT1, VAMP7, DDIT3, DAPK1, ATG12, CAPN1, ITGA3) were found to be significantly associated with overall survival (OS) (shown in Fig. 3a). Next, multivariate cox regression was performed to select the proper ARGs from these 14 genes. After calculating, eight ARGs (ATG5, TP73, BECN1, SIRT1, VAMP7, DAPK1, ATG12, CAPN1) was selected (shown in Table 2). Among these eight genes, BECN1, DAPK1 and CAPN1 played protective roles (HR<1), while the other five genes were risk factors in survival (HR>1). According to the eight genes expressions and their coefficient [18], we then calculated the risk score (= ${\sum}_{n=1}^j\  Coefj\ast Xj$, with Coef j indicating the coefficient and Xj representing the relative expression levels of each ARG standardized by z-score) of each patient and used the median risk score value as a cutoff point for classifying the EAC patients into a high-risk group (*n* = 39) and a low-risk group (*n* = 39), respectively. Patients in the high-risk group obviously had a shorter overall survival time than patients in the low-risk group (median time = 1.10 years vs. 1.752 years, *p* < 0.001, Fig. 3b).

**Table 2:** ARGs associated with prognosis

Gene name	coefficient	HR	95%CI	*P*-value
ATG5	1.639	5.149	1.79–14.791	0.002
TP73	0.461	1.586	0.866–2.905	0.136
BECN1	−1.135	0.321	0.106–0.978	0.046
SIRT1	0.680	1.974	0.859–4.536	0.109
VAMP7	1.647	5.193	1.716–15.716	0.004
DAPK1	−0.453	0.636	0.428–0.945	0.025
ATG12	1.758	5.801	1.201–28.024	0.029
CAPN1	− 0.929	0.395	0.210–0.743	0.004

Eight ARGs were related with OS and used to calculate the risk score to classify the tumor patients into high and low risk groups

Risk score = ∑(exp _i_·coef _i_) exp.: ARGs expression level, coef: coefficient

### Prognostic hazard curves

**Fig. 4:**
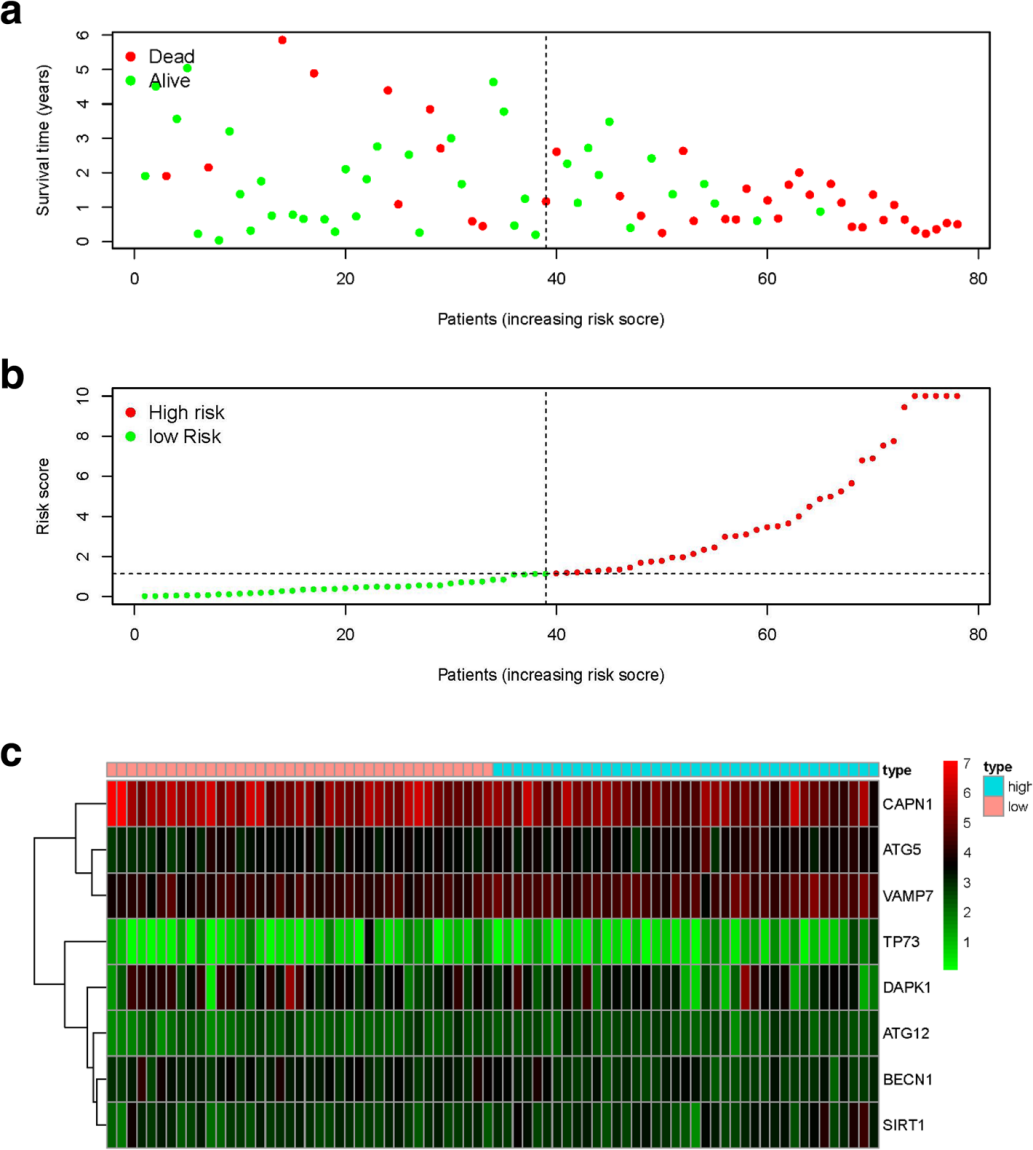
Risk score analyses of high and low risk groups in tumor patients. **a** Risk score scatter plot of high risk and low risk. Red dots represented the dead patients and green represented the alive. With the increase of risk score, more patients died. **b** The dotted line indicates the individual inflection point of the risk score curve, by which the patients were categorized into low-risk and high-risk groups. Risk score high risk (red) and low-risk (green). **c** Risk score heatmap of eight ARGs. The colors from green to red indicate the expression level from low to high

All the tumor patients were divided into high-risk group or low-risk group. As the risk score increased, the patients’ risk increased, and the survival time decreased (Fig. 4a-b). The risk heatmap clearly showed CAPN1 was down-regulated in high-risk group, implying a tumor-suppressor role. However, VAMP7 was up-regulated in high -risk group and this implied it was a tumor-promoting role.

### Independent risk factors of OS

**Fig. 5:**
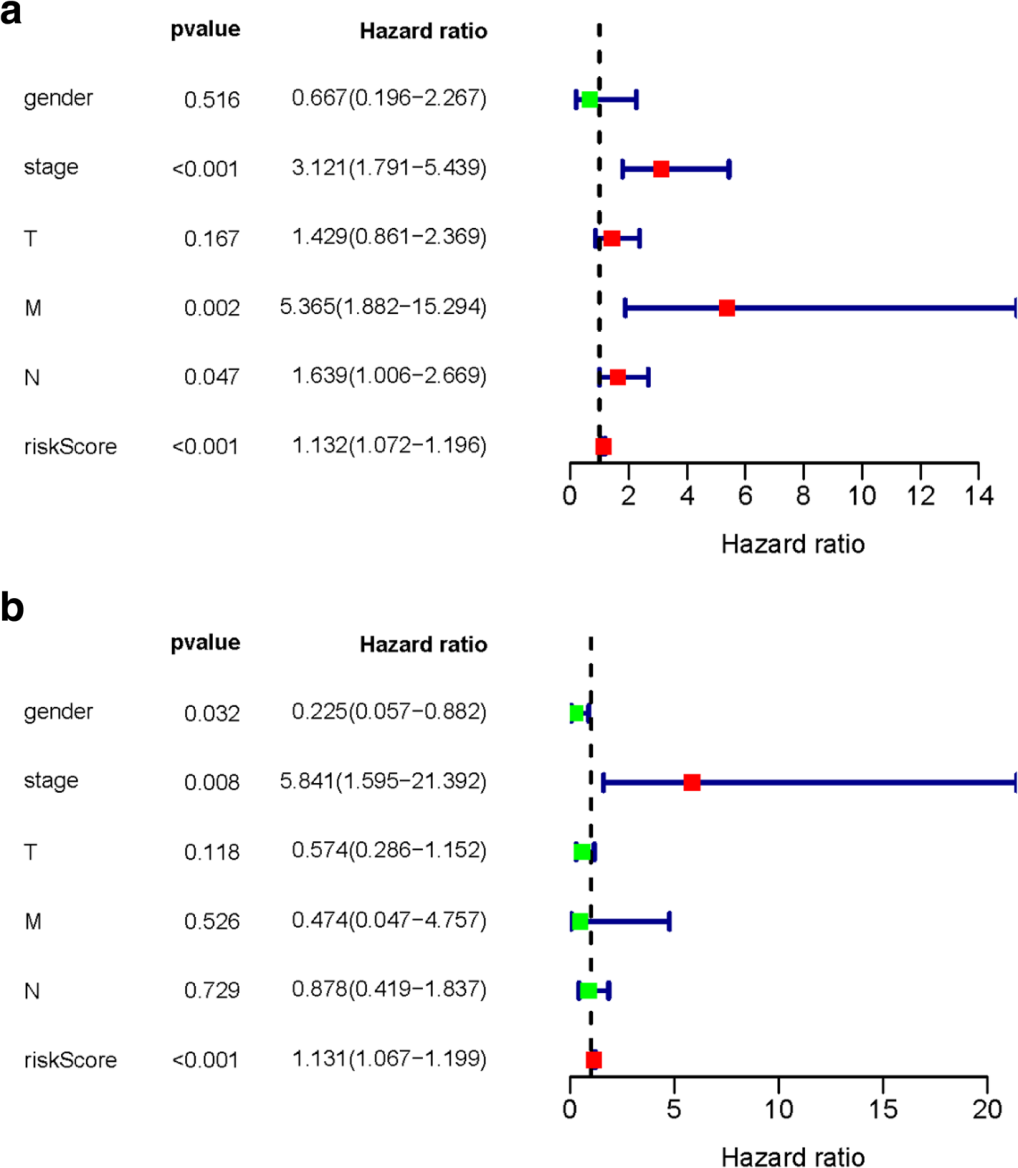
Forest plots of prognostic risk factors **a** univariate cox regression forest plot. **b** Multivariate cox regression forest plot of independent risk factors

We combined the risk score with clinical data in EAC patients. Then we performed univariate cox and multivariate cox regression analyses to investigate the independent risk factors for OS. The univariate cox analysis showed that tumor stage, M (metastasis), N (lymph nodes) and risk score were correlated with OS (*P* < 0.001, *P* = 0.002, *P* = 0.047, *P* < 0.001, respectively) (Fig. 5a). Multivariate cox regression showed gender (HR = 0.225, *P* = 0.032), stage (HR = 5.841, *P* = 0.008) and risk score (HR = 1.131, *P* < 0.001) were independent risk factors for survival (Fig. 5b).

### Model development for predicting the survival

**Fig. 6:**
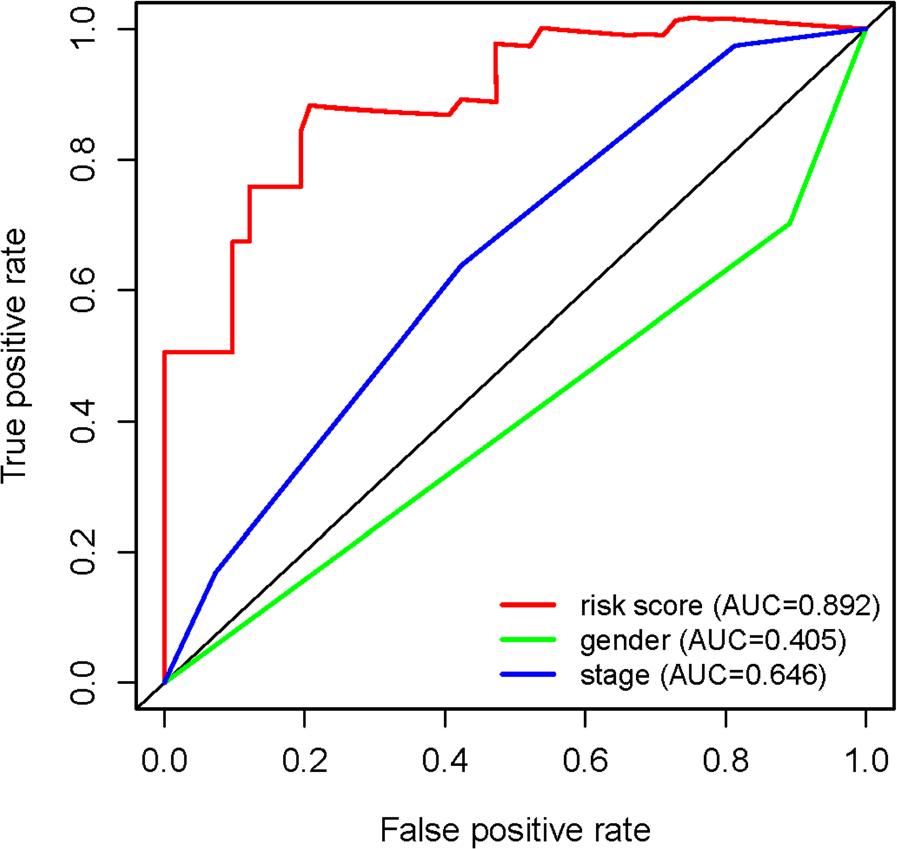
ROC curves of predicting survival. AUC: area under curve. The bigger AUC, the more accurate it predicts

In order to provide an approach to predict the survival, we constructed the ROC curve using the independent risk factors associated with OS (gender, stage and risk score). In addition, we assess the feasibility using the area under curve (AUC) values. The risk score curve showed better ability to predict the survival risk (AUC = 0.892) than other indicators (Fig. 6).

### Clinical correlation analysis

**Fig. 7:**
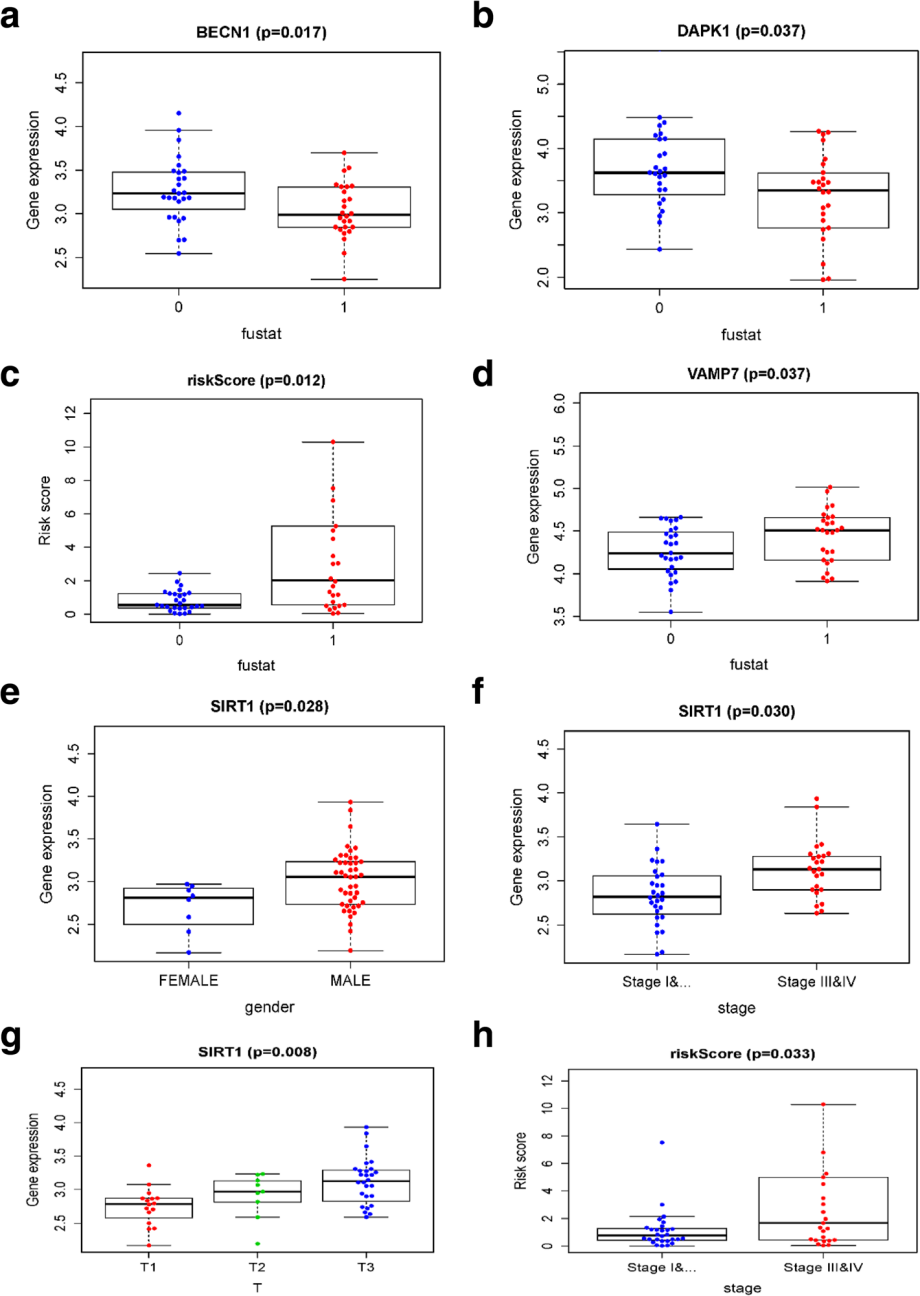
Correlations between ARGs and clinical features. 0 represented the alive, 1 represented the dead in the (**a**-**d**)

In order to explore the relationships between the prognostic eight ARGs and clinical features, we calculated the correlations using the t-test or Kruskal-Wallis test. We found BECN1, DAPK1, VAMP7 and risk score were significantly associated with survival status (all *P* values < 0.05). Among these, BECN1 and DAPK1 expressions were higher in the alive patients, implying their protective roles (Fig. 7a-d). We also found SIRT1 was significantly associated with gender, tumor stage and T (primary tumor) and its expression levels increased with time (Fig. 7e-g). In addition, risk score was also associated with tumor stage (Fig. 7h).

### Experimental validation

**Fig. 8:**
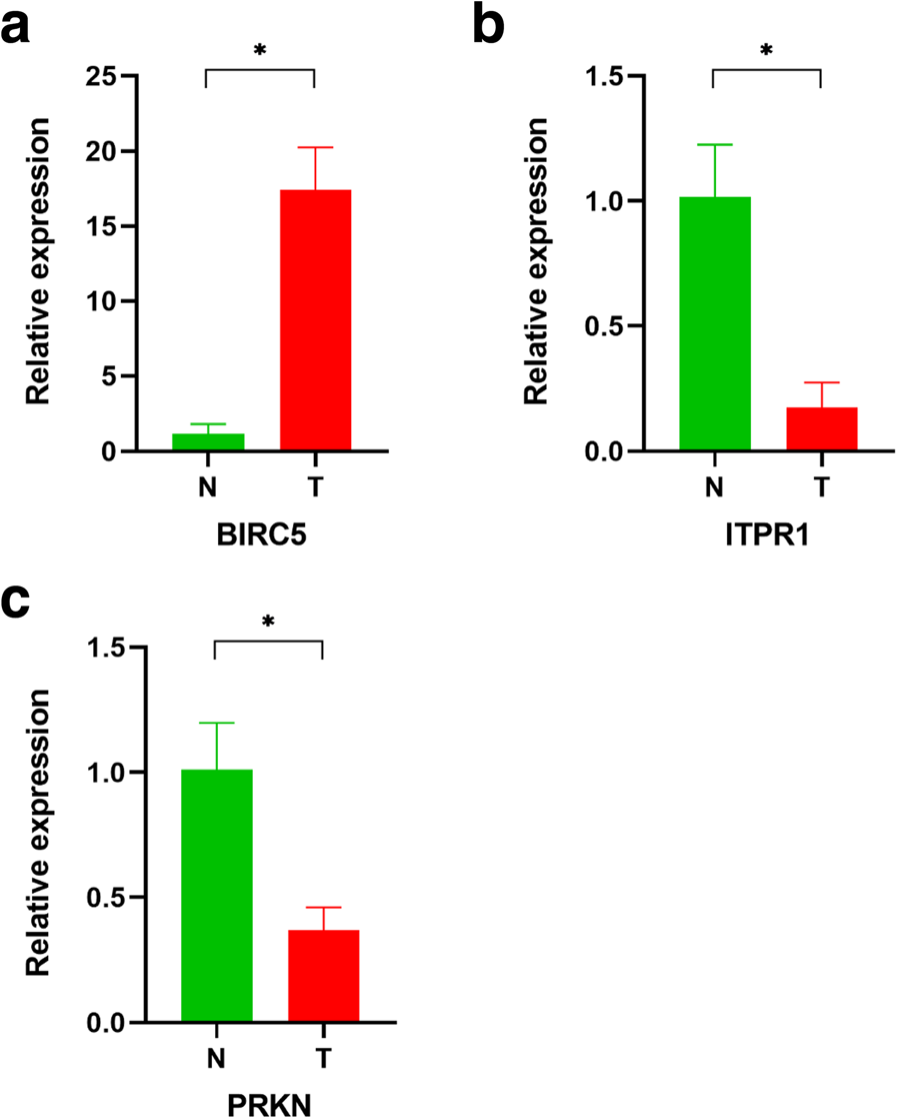
ARsGS expression levels in normal and EAC tissues. The BIRC5 was overexpressed and the ITPR1, PRKN were downregulated in the EAC tissues compared with the normal esophageal mucosal tissues. N: normal tissue, T: tumor. *: significant difference (*P* < 0.05)

According to the screening and validation steps as described above, we selected the four most significant ARGs (BIRC5, CDKN2A, ITPR1,PRKN) from the 30 significantly different genes according to the *P* (<0.0001) and FDR values (<0.0001), and GAPDH was set as an internal reference. The experiment results showed the BIRC5 was overexpressed in the EAC tissues and the ITPR1 and PRKN were downregulated in the normal esophageal mucosal tissues. These results were shown in Fig. 8a-c.

## Discussion

Eukaryotic cells rely on mainly two metabolic processes to maintain cellular hemostasis by eliminating damaged proteins and organelles: the ubiquitin-proteasome system and autophagy [19]. Numerous studies have pointed out autophagic dysfunctions were related with various diseases such as infections, neurodegeneration and tumors [20–22]. Autophagy is a double-edged sword in tumorigenesis, which can suppress or promote tumor development in a context-dependent manner, including the tumor microenvironments and patients’ clinical features. Our study showed 30 SD-ARGs between EAC and normal groups. Among these genes, 6 ARGs were down-regulated and 24 ARGs were up-regulated in EAC. In our experiment, we verified some candidate ARGs from the 30 ARGs and the results demonstrated BIRC5 was overexpressed and the ITPR1, PRKN were downregulated in the EAC tissues compared with the normal esophageal mucosal tissues. These findings were consistent with the results of the TCGA public database. By GO and KEGG enrichment analyses, we discovered these SD-ARGs were mainly enriched in the cellular apoptotic signaling pathway. We also found 8 ARGs had prognostic values, in which the BECN1, DAPK1 and CAPN1 played a protective role in survival. In addition, combination of ARGs and clinical data provided a new method to explore independent survival risk factors, which may be crucial to evaluate prognosis and improve the survival chances under appropriate interventions.

Several studies have strongly supported the associations and their distinct expressions levels between autophagic gene and EAC [23–25]. CDKN2A (also known as p16), an autophagic gene, mainly regulates the G1/S cellular cycle process, which is known as a tumor suppressor gene [26, 27]. Hardie L J et al. found CDKN2A hypermethylation was ubiquitous in EAC compared with normal tissue and had protective roles in the molecular progression of Barrett’s epithelium to EAC, which implicated that CDKN2A was in active state when tumorigenesis [23]. Gockel I et al. provided evidence that higher CXCR4 expression was associated with malignant transformation in EAC [24]. This was consistent with our result that CXCR4 expression was higher in EAC than normal tissue. The interaction of CXCR4 and its ligand SDF-1α, which mediates the activation of phosphatidylinositol 3-kinase (PI3K) and Akt pathways, resulting in cell proliferation [28, 29]. These demonstrated the trend to a less favorable outcome associated with an increased expression of CXCR4 in EAC, although the significant survival correlation was not observed in our study. Our results demonstrated HDAC1 expression was higher in EAC compared with normal control. This is in line with previous work by Langer R et al., revealing HDAC1 had higher expression, especially HDAC2, and was associated with aggressive tumor behavior in EAC [25]. In addition, in vitro studies have shown that high HDAC activity leads to tumor dedifferentiation and enhanced tumor cell proliferation [30]. Hence fore, high HDAC expression may represent a surrogate marker for aggressive tumor behavior in EAC. A promising aspect from HDAC was that HDAC inhibitors have been shown to act as radiosensitizers in a variety of cancer cell lines [31], so HDAC inhibitors might be extremely useful for chemotherapeutic or radio chemotherapeutic combination therapies for EAC. Drugs that target ARGs can be more effective as a result of regulating specific facets of cellular activities. As previously discussed, the CXCR4 inhibition with AMD3100 leads to a significant reduction of esophageal cancer growth and metastases [32]. Other therapeutic strategies utilizing the autophagy include the knockdown of the up-regulation ARGs in EAC. For example, eliminating or silencing the expression CXCR4 and HDAC will have a profound impact in EAC accompanied by long-lasting tumor suppression effect theoretically.

Another crucial mechanism of cell death is apoptosis that involves the activation of catabolic enzymes. The intricate details between autophagy and apoptosis trigger a pivotal crosstalk in the tumor suppression [33]. In our study, GO and KEGG analyses demonstrated SD-ARGs were mainly enriched in the cellular apoptotic pathways, and these results can be confirmed in other studies [34, 35]. Shimizu S et al. reported the BCL2 autophagic family members (BAX, BAK1) not only acted as messages of apoptotic signal converge, but also regulated apoptosis, which showed a good agreement with our results [34]. The interaction between autophagy and apoptosis is mediated by different molecular in EAC microenvironment. In an experiment by Ma Z et al., they revealed that BNIP3, an autophagic gene, could induce esophageal cancer cell apoptosis in hypoxia and autophagic inhibitor 3-methyladenine (3-MA) could augment BNIP3-induced cell apoptosis and death [36]. His study suggested autophagy and apoptosis played the opposing roles in esophageal cancer. In addition, cleavage of Beclin-1 could induce proapoptotic factors release from mitochondria and enhance apoptosis, as well as inhibit autophagic function [37]. The results are consistent with our GO enrichment analysis which showed ARGs were also involved in the mitochondria-related process. These studies added strength to the close correlation between autophagy and apoptosis. However, a further mechanistic understanding of the relation for validation is necessary in EAC.

We conducted cox regressions to identify prognostic ARGs and the results showed ATG5, SIRT1, DAPK1 and other five genes were associated with survival. The ATG5 gene encodes autophagy protein 5 (Atg5), which could combine with Atg12/ Atg16 to form complex involving the process of autophagy [38]. The utility of genes as biomarker has been demonstrated and a number of genes have been reported. In a work by Yang PW et al., increased ATG5 expression was observed in esophageal cancer compared with normal tissue [39]. His group also found higher ATG5 expression tumor group had worse prognosis including the overall survival (OS) and progression-free survival (PFS). This result was consistent with our study, implying the ATG5 was a high-risk gene. BECN1 gene (coding for Beclin 1), exerted a crucial role in autophagosome formation though interacting with Vps34 [40, 41]. Weh KM et al. found Beclin-1 expression loss occurred more frequently in EAC patients compared with controls (49.0% vs 4.8%) [8]. Additionally, Beclin 1 expression level was negatively correlated with EAC histologic grade and stage (*P* < 0.005). Consistently with our study, they also demonstrated the increase in Beclin 1 could cause long-term survival, which implied BECN1 was a tumor suppressor gene and may act as a prognostic biomarker. SIRT1 has function of activating stress response, maintaining genomic integrity and involving the apoptosis and tumorigenesis [42]. Ma M C et al. reported SIRT1 was an independently survival risk factor in esophageal cancer and its overexpression was associated with worse OS (HR = 1.776, *P* = 0.009) and disease-free survival (DFS) (HR = 1.642, *P* = 0.017) [43]. These prognostic ARGs may be useful for early detection and might be a valid strategy to increase the survival chances.

Further analysis of our study showed that risk score was an independent risk factor of prognosis, which suggested autophagy genes could serve as an accurate survival indicator. Patients with high risk score exhibited obvious worse prognosis. ROC curve showed risk score associated with ARGs was the most important variable in predicting the EAC survival, implying its significant potential to be used as a prognostic biomarker. Therefore, more precise individualized treatment strategies for EAC patients with high risk scores should be established. At last, we evaluated the relations between ARGs and patients’ clinical features. The results showed BECN1, DAPK1, VAMP7 and risk score were significantly associated with survival status (all *P* values < 0.05). The significant downregulations of BECN1 and DAPK1 in EAC patients compared with normal control implied that they played protective effects. Whereas the VAMP7 was conferred an increased risk. Moreover, the level of SIRT1 increased with the increasing tumor stage and primary tumor, which indicated ARGs were involved in the progression of EAC and it is essential to increase our understanding of the pathways between autophagy and clinical features.

The strength of our study is that we performed a systematic analysis of autophagic genes from national database, which provided a robust statistical support. While up- or down-regulation of ARGs were explored in this study, it should also be noted that they don’t provide a measure of detecting the overall autophagy levels in EAC tissues. Meanwhile, there are also some limitations. Firstly, the clinical information downloaded from the TCGA was incomplete, especially the therapy, which could be helpful to know whether ARGs are biomarkers of treatment. Secondly, the mechanisms how ARGs modulate the process of EAC were unclear. Lastly, the prognostic model needs to be verified in a large-scale and multicenter clinical cohort. Notwithstanding its limitations, this study does provide a comprehensive overview of ARGs profile in EAC and these limitations can be solved if there are enough data in the future.

## Conclusion

In conclusion, we identified differentially expressed ARGs that could involve in the process of EAC. These ARGs have great potential in diagnostic and prognostic biomarkers and therapeutic targets in EAC patients. Further validations are necessary to confirm the findings of our study.

## Supplementary information

**Additional file 1: Supplementary Table S1.** Primers design and their sequences of ARGs

## Data Availability

All data were available in TCGA database (https://portal.gdc.cancer.gov). And all the experimental data analyzed and displayed in the present manuscript are available from the corresponding author upon reasonable request.

## Abbreviations

EAC: Esophageal adenocarcinoma
ARGs: Autophagy-related genes
OS: Overall survival
BE: Barrett’s esophagus
GERD: Gastroesophageal reflux disease
TCGA: The Cancer Genome Atlas
HADb: Human Autophagy Database
ROC: Receiver Operating Characteristic
SD-ARGs: Significantly different autophagy-related genes
GO: Gene Ontology
BP: Biological process
CC: Cellular component
MF: Molecular function
KEGG: Kyoto Encyclopedia of Genes and Genomes
AUC: Area under curve
cDNA: complementary DNA
gDNA: genomic DNA
PCR: Polymerase chain reaction
GAPDH: Glyceraldehyde-3-phosphate dehydrogenase
PI3K: Phosphatidylinositol 3-kinase
DFS: Disease-free survival
