# Supplementary material for: Profiles of autophagy-related genes in esophageal adenocarcinoma
Source: BMC Cancer. 2020 Oct 1;20:943. doi: 10.1186/s12885-020-07416-w (PMC7528598; doi:10.1186/s12885-020-07416-w)
Supplement: Supplementary file 1 — Additional file 1: Supplementary Table S1. Primers design and their sequences of ARGs [file 12885_2020_7416_MOESM1_ESM.docx]

**Supplementary Table S1 Primers design and their sequences of ARGs**

| **Number** | **Gene Primer** | **Base sequence(5'to 3')** | **base number** | **Purification method** |
| --- | --- | --- | --- | --- |
| 1 | APOL1-F1 | TCAAAGTAAGCCCCTCGGTG | 20 | tPAGE |
| 2 | APOL1-R1 | TCATTTGTCTTGCAAGGTTG | 20 | tPAGE |
| 3 | ATIC-F1 | GTTGTTGCCTGCAATCTCTA | 20 | tPAGE |
| 4 | ATIC-R1 | TGGACACCACCACATAGTCC | 20 | tPAGE |
| 5 | BAK1-F1 | ATCAGCAGGAACAGGAGGCT | 20 | tPAGE |
| 6 | BAK1-R1 | AAGTACTCATAGGCATTCTC | 20 | tPAGE |
| 7 | BAX-F1 | GATGATTGCCGCCGTGGACA | 20 | tPAGE |
| 8 | BAX-R1 | AACCACCCTGGTCTTGGATC | 20 | tPAGE |
| 9 | BCL2L1-F1 | GTGAATGGAGCCACTGGCCA | 20 | tPAGE |
| 10 | BCL2L1-R1 | GTTCCCATAGAGTTCCACAA | 20 | tPAGE |
| 11 | BID-F1 | CAGCTCAGGAACACCAGCCG | 20 | tPAGE |
| 12 | BID-R1 | GCGTAGGTTCTGGTTAATAA | 20 | tPAGE |
| 13 | BIRC5 -F1 | CTTCAAGGAGCTGGAAGGCT | 20 | tPAGE |
| 14 | BIRC5 -R1 | TCCTAAGACATTGCTAAGGG | 20 | tPAGE |
| 15 | BNIP3-F1 | TGGCGCCATGTCGCAGAACG | 20 | tPAGE |
| 16 | BNIP3-R1 | GCTGTCACAGTGAGAGCTCT | 20 | tPAGE |
| 17 | CASP1-F1 | GCTCAGAAGGGAATGTCAAG | 20 | tPAGE |
| 18 | CASP1-R1 | CCCAGATTTTGTAGCAGCAT | 20 | tPAGE |
| 19 | CDKN2A-F1 | TTCGGCTGACTGGCTGGCCA | 20 | tPAGE |
| 20 | CDKN2A-R1 | AGCACCACCAGCGTGTCCAG | 20 | tPAGE |
| 21 | CXCR4-F1 | ATCGTCCACGCCACCAACAG | 20 | tPAGE |
| 22 | CXCR4-R1 | AGATATATCTGTCATCTGCC | 20 | tPAGE |
| 23 | DDIT3-F1 | CTGGTATGAGGACCTGCAAG | 20 | tPAGE |
| 24 | DDIT3-R1 | AGGTGCTTGTGACCTCTGCT | 20 | tPAGE |
| 25 | FADD-F1 | CACGACCTGCTGCGGCGCGT | 20 | tPAGE |
| 26 | FADD-R1 | ATGCTGTCGATCTTGGTGTC | 20 | tPAGE |
| 27 | GABARAPL1-F1 | TCCAGTACAAGGAGGACCAT | 20 | tPAGE |
| 28 | GABARAPL1-R1 | AGGGCACTAGGTACTTCCTC | 20 | tPAGE |
| 29 | HDAC1-F1 | CGGAGTACAGCAAGCAGATG | 20 | tPAGE |
| 30 | HDAC1-R1 | GAAGCCAGATGCCTCGGACT | 20 | tPAGE |
| 31 | HSP90AB1-F1 | TACCAAAGTGATCCTCCATC | 20 | tPAGE |
| 32 | HSP90AB1-R1 | TCTGCCTCATCATCACTAAT | 20 | tPAGE |
| 33 | IKBKE-F1 | TGACAGACTTCGGCGCTGCC | 20 | tPAGE |
| 34 | IKBKE-R1 | ACAAGGTCACTCCAATGCTC | 20 | tPAGE |
| 35 | IL24-F1 | AACTGTGGGAAGCCTTCTGG | 20 | tPAGE |
| 36 | IL24-R1 | CAGAGTCCTGACTTCAACTG | 20 | tPAGE |
| 37 | IRGM-F1 | GTATGACTTCATCATGGTTG | 20 | tPAGE |
| 38 | IRGM-R1 | GAGGTATTTCTCATGGCAGG | 20 | tPAGE |
| 39 | ITGA3-F1 | CAGCAGCGCTACCTGCTCCT | 20 | tPAGE |
| 40 | ITGA3-R1 | GTCACTCCAAGCCACATGTC | 20 | tPAGE |
| 41 | ITGB4-F1 | CAGGACACAGGACTACCCGT | 20 | tPAGE |
| 42 | ITGB4-R1 | AGCAGCTCCACGATGTTGGA | 20 | tPAGE |
| 43 | ITPR1-F1 | GCGGAGCAGGGTATTGGAAC | 20 | tPAGE |
| 44 | ITPR1-R1 | GCATTCCGCAACCTACTTCG | 20 | tPAGE |
| 45 | NKX2-3-F1 | GCGAGACTCGTGCAGCGAGC | 20 | tPAGE |
| 46 | NKX2-3-R1 | GCGTTCCAGCTCGAAGACCT | 20 | tPAGE |
| 47 | PPP1R15A-F1 | CTACTCATGATCCGGACCCT | 20 | tPAGE |
| 48 | PPP1R15A-R1 | ATCCCGAGCAAGCTGCTCCCA | 21 | tPAGE |
| 49 | PRKN-F1 | CAGCTCAAGGAGGTGGTTGC | 20 | tPAGE |
| 50 | PRKN-R1 | TCGCCTCCAGTTGCATTCAT | 20 | tPAGE |
| 51 | RGS19-F1 | CCCTTCAATGTCCAGTCATG | 20 | tPAGE |
| 52 | RGS19-R1 | TGGCACATACTTCACAGCTG | 20 | tPAGE |
| 53 | SPHK1-F1 | AAGGGCAAGGCCTTGCAGCT | 20 | tPAGE |
| 54 | SPHK1-R1 | CGTTCACCACCTCGTGCATC | 20 | tPAGE |
| 55 | TNFSF10-F1 | ATCGTGATCTTCACAGTGCT | 20 | tPAGE |
| 56 | TNFSF10-R1 | GGTCCCAATAACTGTCATCT | 20 | tPAGE |
| 57 | VEGFA-F1 | CTTGGGTGCATTGGAGCCTT | 20 | tPAGE |
| 58 | VEGFA-R1 | ATTGGATGGCAGTAGCTGCG | 20 | tPAGE |
| 59 | VMP1-F1 | AGTGCATCAACAGTATGTGC | 20 | tPAGE |
| 60 | VMP1-R1 | TGTGGACCCAGATAAAGCAG | 20 | tPAGE |
| 61 | ATG12-F1 | CAGCTTCCTACTTCAATTGC | 20 | tPAGE |
| 62 | ATG12-R1 | TCTTGGTGTCGCCAGCAGGT | 20 | tPAGE |
| 63 | ATG5 -F1 | ACTCATGGAATATCCTGCAG | 20 | tPAGE |
| 64 | ATG5 -R1 | TCTTCAGGATCAATAGCAGA | 20 | tPAGE |
| 65 | BECN1-F1 | ACTCCTCGCCAGGATGGTGT | 20 | tPAGE |
| 66 | BECN1-R1 | TGTCTGGCCCGACATGATGT | 20 | tPAGE |
| 67 | CAPN1-F1 | AAGGCAACGAGTTCTGGAGC | 20 | tPAGE |
| 68 | CAPN1-R1 | TGATCTGGTAGAGGTCACTG | 20 | tPAGE |
| 69 | DAPK1-F1 | TATGAACCTCTTGGTCTTGA | 20 | tPAGE |
| 70 | DAPK1-R1 | TCAAATTCGTAGTTGACAGC | 20 | tPAGE |
| 71 | SIRT1-F1 | TGACTGTGAAGCTGTACGAG | 20 | tPAGE |
| 72 | SIRT1-R1 | ACAATGAGGAGGTCAACTTC | 20 | tPAGE |
| 73 | TP73-F1 | ATGAGGACCACTACCGGGAG | 20 | tPAGE |
| 74 | TP73-R1 | TGAAGGTAGTACGTGTCCTC | 20 | tPAGE |
| 75 | VAMP7-F1 | CTATCCTTGCCAAACATGCT | 20 | tPAGE |
| 76 | VAMP7-R1 | GAGAACTCGCTATTCATGGC | 20 | tPAGE |
